# Rewiring the luteal microenvironment: hemodynamic and molecular insights into eCG-supported CL development in indigenous White Lamphun cattle

**DOI:** 10.1530/RAF-25-0086

**Published:** 2025-12-01

**Authors:** Jakree Jitjumnong, Punnawut Yama, Molarat Sangkate, Assawadet Suriard, Wichayaporn Butmata, Noppanit Daoloy, Anukul Taweechaipaisankul, Songphon Buddhasiri, Chih-Jen Lin, Yanli Zhang, Tossapol Moonmanee

**Affiliations:** ^1^ Department of Animal and Aquatic Sciences, Faculty of Agriculture, Chiang Mai University, Chiang Mai, Thailand; ^2^ Functional Feed Innovation Center, Faculty of Agriculture, Chiang Mai University, Chiang Mai, Thailand; ^3^ Department of Animal Science and Fishery, Faculty of Sciences and Agricultural Technology, Rajamangala University of Technology Lanna, Phitsanulok, Thailand; ^4^ Department of Biology, Faculty of Science, Chulalongkorn University, Bangkok, Thailand; ^5^ Faculty of Veterinary Medicine, Chiang Mai University, Chiang Mai, Thailand; ^6^ Centre for Reproductive Health, Institute for Regeneration and Repair (IRR), University of Edinburgh, Edinburgh, UK; ^7^ Jiangsu Livestock Embryo Engineering Laboratory, Nanjing Agricultural University, Nanjing, PR China

**Keywords:** corpus luteum development, hemodynamics, luteal angiogenic gene expression, white lamphun cattle

## Abstract

**Abstract:**

This study evaluated the effects of low-dose equine chorionic gonadotropin (eCG) supplementation on follicular dynamics, ovulation, corpus luteum (CL) development, luteal hemodynamics, and angiogenesis-related gene expression in indigenous White Lamphun cows under a short-duration estrous synchronization protocol. Twenty multiparous cows were randomly allocated into two groups: a control group (*n* = 10) and an eCG-treated group (*n* = 10) receiving 200 IU eCG intramuscularly at CIDR removal (day 0). Daily B-mode ultrasonography revealed significantly faster dominant follicle growth and larger preovulatory follicle (POF) diameters in the eCG group (*P* < 0.05). CL development assessed on days 4 and 11 post-ovulation (CL 4 and CL 11) showed greater CL diameter, area, and volume in the eCG group (*P* < 0.01 and *P* < 0.001, respectively). Color Doppler imaging indicated improved luteal hemodynamics, with a higher colored area/total CL area ratio on both CL 4 (*P* < 0.05) and CL 11 (*P* < 0.01). Plasma progesterone (P4) concentrations were significantly elevated at CL 11 (*P* < 0.05), while preovulatory estradiol (E2) levels were also higher in the eCG group (*P* < 0.05). Gene expression analysis of luteal tissues on CL 11 revealed significant upregulation of *NOTCH4*, *JAG1*, and *CD300LG* (*P* < 0.05), whereas *NOS3* and *MMP9* did not differ significantly between groups (*P* > 0.05). These results indicate that low-dose eCG enhances follicular development, CL function, and luteal angiogenesis, providing a promising strategy to improve fertility in *Bos indicus* cattle.

**Lay summary:**

This study tested whether a small dose of equine chorionic gonadotropin (eCG), a hormone naturally produced during pregnancy in horses, can enhance ovarian function in native Thai cattle. We added eCG to a short estrous synchronization program, a hormone-based protocol used to align the timing of ovulation among cows within a few days, and evaluated key reproductive processes: egg release (ovulation), development of the CL, a temporary gland formed after ovulation that secretes progesterone (P4), a hormone essential for establishing and maintaining early pregnancy, and blood flow to the CL. We also measured hormone levels and the activity of genes involved in forming new blood vessels. Cows that received eCG showed stronger ovarian activity, including larger preovulatory follicles, more robust CL development, better ovarian blood flow, and higher P4 levels. Genes related to vascular growth were also more active. These findings indicate that low-dose eCG supports CL function and ovarian physiology and may help improve the efficiency of breeding management in local cattle.

## Introduction

Reproductive efficiency is fundamental to sustainable livestock production, particularly in tropical regions where heat and nutritional stress compromise fertility. Indigenous breeds such as the White Lamphun (*Bos indicus*) are well adapted to harsh, low-input systems but often exhibit irregular estrous cycles, delayed ovulation, and low conception rates. Improving their reproductive performance is therefore critical for enhancing productivity and smallholder sustainability. Estrous synchronization protocols combining hormonal treatments have been widely applied to improve fertility in cattle, though their application in White Lamphun cows remains limited. Short-term progesterone-based regimens using controlled internal drug release (CIDR) devices with gonadotropin-releasing hormone (GnRH) and prostaglandin F2α (PGF2α) are effective for inducing synchronized ovulation and improving conception rates in *Bos indicus* breeds (Baruselli *et al.* 2004, Bó & Baruselli 2014, Neto *et al.* 2024). Building on this approach, the present study evaluated whether supplementing the synchronization protocol with low-dose equine chorionic gonadotropin (eCG) could enhance ovarian function in indigenous cattle.

In cattle, eCG supplementation during synchronization protocols enhances follicular dynamics and corpus luteum (CL) development, increasing dominant follicle (DF) diameter, ovulation rate, and CL size with elevated progesterone (P4) secretion (Bó & Mapletoft 2014, Neto *et al.* 2024). These effects reflect the ability of eCG to promote preovulatory follicle maturation and luteinization, thereby improving the endocrine milieu for conception and pregnancy. The CL is critical for reproductive success through sustained P4 secretion, which depends on adequate blood perfusion (Niswender *et al.* 2000, Lüttgenau & Bollwein 2014). Angiogenesis supplies nutrients, supports steroidogenesis, and links vascular development with endocrine competence (Reynolds & Redmer 1995, Lüttgenau & Bollwein 2014). Color Doppler ultrasonography now enables non-invasive assessment of luteal blood flow, which correlates positively with P4 concentration, embryo development, and fertility (Ginther *et al.* 2007, Takasaki *et al.* 2009, De Tarso *et al.* 2017). Despite these insights, the physiological effects of eCG on follicular development, luteal function, and hemodynamics remain underexplored in indigenous breeds such as White Lamphun cattle, which are vital to northern Thailand’s sustainable livestock systems.

Beyond morphological and functional traits, luteal activity is tightly regulated by angiogenesis and vascular remodeling, processes governed by specific molecular pathways. Key genes such as *NOTCH4* and *JAG1* mediate endothelial communication and vessel stabilization via the Notch signaling pathway (Iso *et al.* 2003, Gridley 2010), while *CD300LG* regulates endothelial adhesion and vascular permeability (Umemoto *et al.* 2013). Additional angiogenic markers, including *NOS3* and *MMP9*, control vasodilation and extracellular matrix remodeling, respectively (Donald *et al.* 2015, Nikanfar & Amorim 2025). Evaluating the expression of these genes offers mechanistic insight into vascular adaptations induced by hormonal stimulation such as eCG. Therefore, this study integrated physiological indicators (follicular growth, CL development, hormone secretion, and hemodynamics) with gene expression profiling to elucidate the effects of low-dose eCG in White Lamphun cows and to inform fertility-enhancing strategies for tropical cattle systems.

## Materials and methods

### Ethical aspects

This study was approved by the Institutional Animal Care and Use Committee (IACUC) of the Faculty of Agriculture, Chiang Mai University under protocol number RAGIACUC028/2567.

### Location

The experiment was conducted at the Beef Cattle Facility, Department of Animal and Aquatic Sciences, Faculty of Agriculture, Chiang Mai University, located within the Agricultural Innovation Research, Integration, Demonstration, and Training Center (AIRIDTC) (Latitude: 18°45′ N; Longitude: 98°55′ E).

### Animals

A total of 20 multiparous indigenous cows (White Lamphun cattle; *Bos indicus*), aged 3–5 years and weighing 228.4 ± 9.8 kg, were used in this experiment. All animals were clinically healthy, showing no signs of infectious or metabolic disease. Before the start of the experiment, the cows were dewormed and vaccinated against hemorrhagic septicemia and foot-and-mouth disease. Body condition scores (BCS) were assessed at the beginning of each synchronization protocol using a 1 to 5 scale, where 1 indicates emaciation and 5 indicates obesity (Ayres *et al.* 2009). The average BCS was 3.0 ± 0.2, indicating that all cows were in moderate condition. The experiment was conducted from mid-January to the end of March 2025. During this period, the cows were housed in an indoor corral at the Beef Cattle Facility, provided with mineral supplementation, free access to water, and maintained under strict sanitary conditions.

### Experimental design

The cows (*n* = 20) were randomly allocated into two experimental groups: a control group without eCG administration (without eCG/control; *n* = 10) and a treatment group with low-dose eCG administration (low-dose eCG; *n* = 10).

### Hormonal synchronization protocol

**Figure 1:**
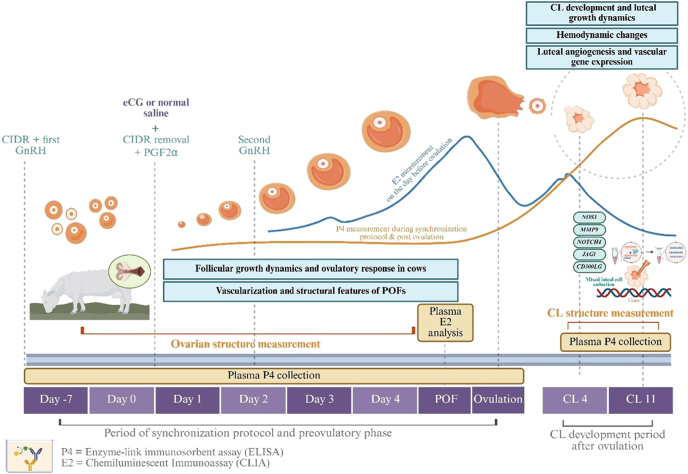
Experimental design and timeline of sample collection and analysis in indigenous White Lamphun cows. The diagram illustrates the hormonal synchronization protocol and timeline of sample collection used in this study. On day-7, a CIDR device was inserted, and the first GnRH injection was administered. On day 0, the CIDR was removed, followed by PGF2α administration and either eCG (low-dose eCG) or normal saline (without eCG/control). A second GnRH injection was given on day 2. Follicular growth dynamics, ovulatory response, and ovarian structure were monitored daily via ultrasonography (B-mode and color Doppler) from day 0 until ovulation. Plasma estradiol (E2) levels were measured on the day before ovulation using chemiluminescent immunoassay (CLIA), while plasma P4 was assessed using enzyme-linked immunosorbent assay (ELISA) at multiple time points: day-7, day 0 through the day of ovulation, and days 4 and 11 after ovulation. On day 11 post-ovulation (CL 11), luteal tissues were collected to evaluate luteal angiogenic and vascular gene expressions, including *NOS3*, *MMP9*, *NOTCH4*, *JAG1*, and *CD300LG*.

In both experimental groups, the estrous synchronization protocol was initiated on day-7 (D-7) with the insertion of an intravaginal device containing 1.38 g of progesterone (Eazi-Breed CIDR, Zoetis Inc., New Zealand), followed by an intramuscular (IM) administration of 10 μg of gonadotropin-releasing hormone (GnRH; Buserelin, Receptal, MSD Animal Health, New Zealand). On day 0 (D0), the progesterone device was removed, and 500 μg of prostaglandin F2α (PGF2α; Cloprostenol, Estrumate, MSD Animal Health, New Zealand) was administered via IM injection. On the same day, the low-dose eCG group received an additional IM injection of 200 IU of equine chorionic gonadotropin (eCG; Folligon^®^, Intervet Limited, Thailand), while the control group received an equivalent volume of sterile saline. On day 2 (D2), both groups received a second IM injection of 10 μg GnRH (Fig. 1). In this study, 200 IU of eCG was defined as a low dose relative to the standard synchronization protocols in *Bos indicus* cattle, which typically use 300–500 IU (Baruselli *et al.* 2004, Bó & Baruselli 2014). This lower dose was specifically selected to reflect the smaller body size and higher gonadotropin sensitivity of indigenous White Lamphun cows, thereby reducing the risk of overstimulation while still effectively supporting follicular and luteal function.

### Ultrasonographic evaluation of follicular development and ovulation timing

The characteristics of the DF and the preovulatory follicle (POF) were meticulously evaluated through daily transrectal ultrasonographic examinations using a B-mode ultrasound scanner equipped with a 5 MHz linear-array transducer (SonoscapeTM, Model E2V, China). High-resolution ultrasonography was employed to monitor the development and structural changes of ovarian follicles throughout the estrous cycle. Follicles with a diameter ≥2 mm, as well as the CL, were routinely measured and recorded on an ovarian ultrasonographic diagram to enable precise tracking of follicular growth and development. Ovarian ultrasonographic scanning was performed daily, beginning on day 0 of the synchronization protocol and continuing until ovulation was confirmed. This approach allowed for continuous monitoring of DF and POF diameters during the critical preovulatory phase, providing valuable data on follicular dynamics. The time of ovulation and ovulation rate were determined by observing the disappearance of a POF with a diameter ≥7 mm between 2 consecutive days of ultrasonographic assessments. This method enabled accurate identification of ovulation timing and quantification of the proportion of animals that successfully ovulated following synchronization.

### Luteal evaluation

CL development was evaluated on days 4 and 11 post-ovulation. To assess ovarian luteal parameters, including CL diameter, area, and volume, the location and diameter of each CL were recorded on an ovarian map, and ultrasonographic images were archived for subsequent analysis. CL diameter was measured using the internal calipers of the ultrasound machine. CL area was calculated as previously described by (Pirokad *et al.* 2022) using the following formula: CL area = *π*/4 × (mean CL diameter)^2^. In cases where a cavity was present, the area was adjusted using the formula: CL area = *π*/4 × (mean CL diameter)^2^ − *π*/4 × (mean CL cavity diameter)^2^, where *π* = 3.1416. CL volume was determined based on the method described by (Tortorella *et al.* 2013, Pirokad *et al.* 2022) using the formula: CL volume = (4/3) × *π* × *r*^3^, where *r* is the CL radius. CL diameter was recorded in centimeters (cm), while CL area and volume were expressed in square centimeters (cm^2^) and cubic centimeters (cm^3^), respectively. CL growth rate was defined as the linear change in CL diameter between days 4 and 11 using the formula: CL growth rate (cm/day) = (CL diameter on day 11 − CL diameter on day 4)/7, calculated in ovulated cows with measurements available at both time points.

### Evaluation of ovarian hemodynamic changes using Doppler ultrasonography

To evaluate hemodynamic changes in ovarian structures, transrectal color Doppler ultrasonography was performed to visualize blood perfusion in the POF and CL. Imaging was conducted using a B-mode ultrasound scanner equipped with a 5.0 MHz linear-array transducer and integrated color Doppler software (SonoscapeTM, Model E2V, China), following protocols described by (Lüttgenau & Bollwein 2014). Vascularized regions within the POF and CL were identified as areas displaying color flow signals, indicative of active blood perfusion. Color Doppler signals were evaluated only in the follicular wall, excluding the antral cavity, to avoid artifacts. Vascularized areas were digitally quantified to assess relative hemodynamic activity, following established protocols (Bollwein *et al.* 2002, Acosta *et al.* 2003, Lüttgenau & Bollwein 2014). Doppler color images were processed using Adobe Photoshop CC (version 2020; Adobe Systems, USA). The Magnetic Lasso Tool was employed to delineate the boundaries of the total structure, the vascularized region (color signal), and any fluid-filled cavities such as the POF antrum or CL cavity. Pixel densities of each outlined region were calculated to determine respective areas. Hemodynamic activity was expressed as the proportion of the vascularized (color Doppler) area relative to the total functional tissue area, using the following formulas: POF vascularity (%) = (colored Doppler area in pixels)/(POF area − POF antrum area in pixels) × 100: CL vascularity (%) = (colored Doppler area in pixels)/(CL area in pixels) × 100: CL tissue vascularity (%) (adjusted for cavity) = (colored Doppler area in pixels)/(CL area − CL cavity area in pixels) × 100. These calculations provided a quantitative assessment of blood flow within ovarian structures, offering valuable insight into tissue perfusion during the periovulatory and luteal phases. This approach enables a detailed evaluation of follicular and luteal hemodynamics, which are critical indicators of ovarian function and reproductive competence.

### Evaluation of circulating E2 and P4 levels

Plasma samples were collected daily from day-7 until ovulation was confirmed in each cow to quantify plasma P4 concentrations. Additional samples were collected on the day of ovulation or when a POF was identified to determine E2 concentrations. Further plasma samples were obtained on days 4 and 11 post-ovulation for subsequent P4 analysis. Immediately after collection, blood samples were centrifuged at 800 ***g*** for 15 min at 4°C to separate the plasma, which was then stored at −20°C until analysis. Plasma hormone concentrations were determined by a certified private diagnostic laboratory (BRIA Laboratory, Thailand) using validated commercial enzyme-linked immunosorbent assay (ELISA) and chemiluminescent immunoassay (CLIA) methods routinely applied for bovine hormone testing for P4 and E2, respectively. The intra-assay coefficients of variation were 13.87% for P4 and 11.4% for E2, with assay sensitivities of 0.02 ng/mL and 10 pg/mL, respectively.

### Transvaginal ultrasound-guided luteal biopsy (OPU-style)

On day 11 post-ovulation, cows were restrained in a chute and received local anesthesia at the lumbosacral junction (2% lidocaine, 1–5 mg/kg). The perineal area was clipped, scrubbed with antiseptic solution, and aseptically prepared. Biopsies were performed using an EMP G30 ultrasound unit with a 5.0 MHz transvaginal linear-array probe and a disposable needle guide (regular OPU guide; CMC BIOTECH CO., LTD, Thailand). The ovary bearing the corpus luteum (CL) was gently stabilized via rectal manipulation to allow accurate positioning of the ovary during ultrasound-guided needle insertion, and a sterile 18 G × 60 cm OPU aspiration/biopsy needle, beveled tip (Pornchai, Thailand; catalog no. 328350) was advanced through the guide under real-time ultrasound into the luteal parenchyma. Using gentle negative pressure (50-mL syringe), 2–3 tissue fragments (∼2–4 mm) were collected from a single puncture track (at most one additional pass through the same tract, if required). Hemostasis was verified ultrasonographically; samples were immediately placed in ice-cold PBS and processed without delay for molecular analyses. Animals were monitored for 48 h; no complications were observed. This standard OPU-style, transvaginal, non-surgical approach follows established bovine ovarian/CL sampling methods (Pieterse *et al.* 1991, Kot *et al.* 1999, Aerts *et al.* 2005, Strüve *et al.* 2013).

### RNA extraction and cDNA synthesis from luteal tissue

Total RNA was extracted from luteal tissue collected on day 11 post-ovulation using RNeasy Micro Kit (Qiagen, Germany) following the manufacturer’s protocol. RNA was eluted in 20 μL of RNase-free water and quantified with a NanoDrop^TM^ One spectrophotometer (Thermo Scientific, USA) by measuring absorbance at 260 and 280 nm. Only samples with acceptable A260/280 ratios (typically between 1.8 and 2.1) were used for downstream applications. For cDNA synthesis, 500 ng of RNA was reverse-transcribed using the Transcriptor First Strand cDNA Synthesis Kit (Roche, Germany). Primers were designed from bovine mRNA sequences (NCBI GenBank; accession numbers in Table 1) using Primer-BLAST under standard parameters, and specificity was verified by BLAST analysis and melt curve validation. The sequences of primers used for each target gene are listed in Table 1. Quantitative real-time PCR (qRT-PCR) was performed in triplicate on a CFX96 Touch Deep Well Real-Time PCR System (Bio-Rad, USA). Each 20 μL reaction contained 100 ng cDNA, 400 nM of each primer, and iTaq™ Universal SYBR® Green Supermix (2X) (Bio-Rad, USA). Gene expression was normalized to glyceraldehyde-3-phosphate dehydrogenase (*GAPDH*) as the internal reference, and relative expression was calculated using the 2^−ΔΔCt^ method (Livak & Schmittgen 2001). To complement Doppler-derived perfusion indices, we pre-specified an angiogenesis-focused gene panel: *NOTCH4* and *JAG1* (endothelial signaling and sprouting), *CD300LG* (endothelial adhesion and permeability), *NOS3* (vasomotor regulation), and *MMP9* (matrix remodeling). This panel was selected to capture vascular sprouting, stabilization, and functional perfusion expected at day 11 CL while ensuring assay feasibility with validated bovine primers and RNA yields from CL biopsies.

**Table 1:** Primers used for quantitative real-time PCR (qRT-PCR).

Genes	Primer sequences (5′-3′)		Product size (bp)	Accession no.
	Forward	Reverse		
*GAPDH*	CAC​CCT​CAA​GAT​TGT​CAG​CA	GGT​CAT​AAG​TCC​CTC​CAC​GA	103	XM_019960295.2
*NOS3*	CCT​CAC​CGC​TAC​AAT​ATC​CT	TGC​TCG​TTG​TCC​AGG​TGC​TTC	197	XM_070788417.1
*MMP9*	GAGAGGGTCGCAATGATG	CTG​GCA​CGG​AGG​TGT​GAT​CTA	159	XM_019972500.2
*NOTCH4*	CAG​GCC​ATC​TCT​GTG​AAA​TTC	GGT​GGC​AGG​TGC​AGT​TGT​CTT	147	XM_070778156.1
*JAG1*	TCC​TAC​ACT​TTG​CTC​GTG​GAG	ACT​TAT​TGC​AGC​CGA​AGC​C	205	XM_019972877.2
*CD300LG*	GAT​GAA​GAG​CCC​GGC​CTC​T	CTT​GTG​CTC​CCA​GGT​TAC​G	144	XM_070773578.1

### Statistical analysis

All statistical analyses were conducted using GraphPad Prism v10.0 (GraphPad Software Inc., USA). Normality of data distribution was evaluated with the Shapiro–Wilk test. For normally distributed variables, group comparisons were performed with unpaired, two-tailed Student’s *t*-tests. When normality was not satisfied, data were either log-transformed to improve distributional assumptions or analyzed using the Mann–Whitney U test. Categorical outcomes, including POF diameter class (<11 mm vs >11 mm) among ovulated cows, ovulatory versus anovulatory proportions, and the distribution of ovulation timing (72, 96, 120 h), were analyzed with two-tailed Fisher’s exact tests or chi-square tests of independence, as appropriate. Results are presented as mean ± SD. Statistical significance was defined as *P* < 0.05, whereas 0.05 ≤ *P* < 0.10 was considered a tendency. Associations among key variables, including follicular growth rate, POF area, CL size and volume, blood perfusion indices, and hormone concentrations, were assessed using Pearson’s correlation coefficients (*r*). Figures (box and column plots with distribution overlays) were generated in OriginPro 2024 (OriginLab Corporation, USA).

## Results

### Follicular growth dynamics and ovulatory response in cows

**Figure 2:**
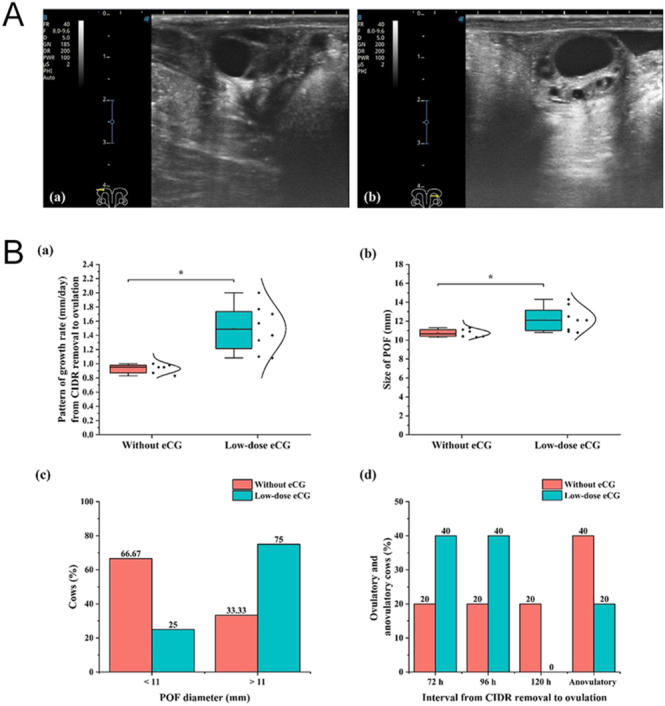
Effect of low-dose eCG treatment on preovulatory follicle growth, size, and ovulatory response in cows undergoing a synchronization protocol. (A) Representative ultrasonographic images of preovulatory follicles (POFs) in cows: (a) without eCG treatment and (b) with low-dose eCG treatment. (B) Comparison between cows treated without eCG and with low-dose eCG in terms of: (a) pattern of POF growth rate (mm/day); (b) size of POF (mm) at ovulation; (c) proportion of ovulated cows with POF diameters <11 mm or >11 mm at ovulation (control: *n* = 6; low-dose eCG: *n* = 8); and (d) distribution of cows based on ovulatory and anovulatory response at specific time points (72, 96, and 120 h) after CIDR removal. All groups originally contained ten cows (without eCG, *n* = 10; low-dose eCG, *n* = 10). For POF diameter analysis (panel c), only ovulated cows were included (without eCG, *n* = 6; low-dose eCG, *n* = 8). Data are presented as box plots with individual data points and normal distribution curve (a, b) and column plot (c, d). * Indicates significant differences (*P* < 0.05).

Ultrasonographic images of the POF are shown in Fig. 2A. Cows treated with low-dose eCG displayed a more prominent and visibly larger follicular structure compared to those without eCG administration (Fig. 2A, panels a and b). Quantitative analysis demonstrated that low-dose eCG significantly increased (*P* < 0.05) the pattern of follicular growth rate that subsequently ovulated as the POF (Fig. 2B(a)). At ovulation, the diameter of the POF was significantly larger (*P* < 0.05) in the eCG group than in the control group (Fig. 2B(b)). Further evaluation of follicular size distribution, calculated only from ovulated cows, showed that in the control group (*n* = 6), 4/6 cows (66.67%) had POF diameters <11 mm, and 2/6 cows (33.33%) had POF diameters >11 mm. In contrast, in the low-dose eCG group (*n* = 8), 2/8 cows (25%) had POF diameter <11 mm, while 6/8 cows (75%) had POF diameters >11 mm (Fig. 2B(c)). Although these differences were not statistically significant (*P* > 0.05), the numerical distribution suggests a shift toward larger POFs in the low-dose eCG group. With respect to ovulatory response, a greater proportion of cows in the eCG group ovulated earlier, with 40% ovulating at both 72 and 96 h after CIDR removal, compared with 20% in the control group at the same time points (Fig. 2B(d)). Anovulatory cows accounted for 40% of the control group but only 20% of the eCG group. While these differences were not statistically significant (*P* > 0.05), the trends suggest that eCG supplementation may improve ovulation synchrony and reduce, though not completely eliminate, anovulatory responses. Collectively, these findings indicate that low-dose eCG supports enhanced DF growth, increases POF size, and may promote more synchronized and reliable ovulatory outcomes in indigenous White Lamphun cows.

### Blood perfusion and structural features of POFs

**Figure 3:**
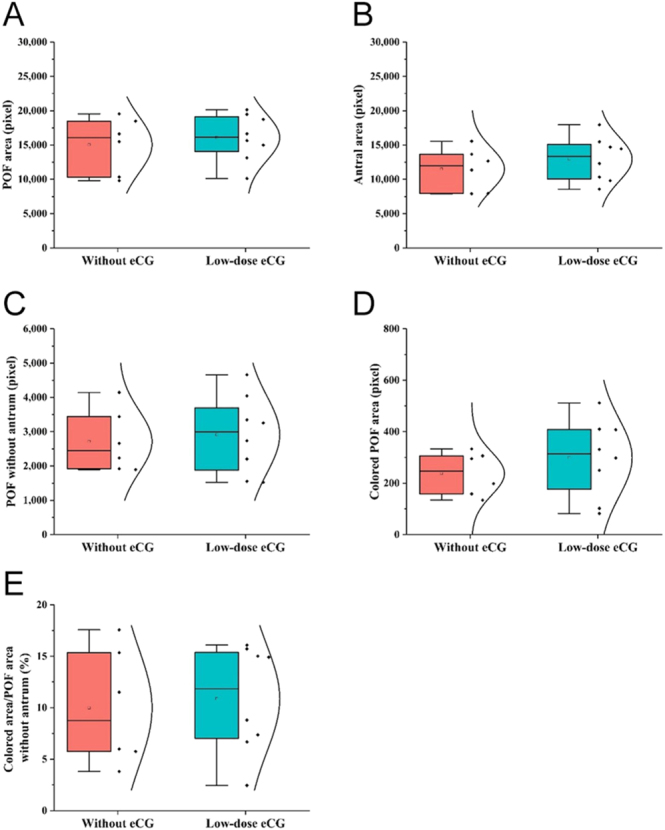
Effects of low-dose eCG treatment on morphological and hemodynamic changes of preovulatory follicles (POFs) in cows. Box plots with individual data points and normal distribution curves compare cows treated without and with low-dose eCG in terms of: (a) POF area (pixel), (b) antral area (pixel), (c) POF without antrum (pixel), (d) colored Doppler area within the follicular wall (pixel), and (e) colored area/POF area without antrum (%). Color Doppler signals detected within the antrum were considered artifacts and excluded from the analysis.

To further characterize the effects of low-dose eCG on follicular development, detailed morphometric and vascular analyses of the POF were conducted (Fig. 3). The total POF area tended to be larger in cows that received low-dose eCG compared with control, although the difference was not statistically significant (*P* > 0.05 for all parameters) (Fig. 3A). Likewise, both the antral area and the POF area without antrum showed higher mean values in eCG-treated cows, but these differences were also non-significant (*P* > 0.05) (Fig. 3B and C). The colored Doppler area, representing blood perfusion within the POF, was numerically greater in the low-dose eCG group (Fig. 3D). When normalized to the POF area without antrum, the relative colored area was higher in eCG-treated cows, suggesting a possible trend toward improved follicular blood perfusion (Fig. 3E). Although none of the morphometric or vascular parameters reached statistical significance, the consistent numerical increases observed across all indices may indicate a mild physiological response to eCG that supports follicular function and maturation.

### CL development and luteal growth dynamics

**Figure 4:**
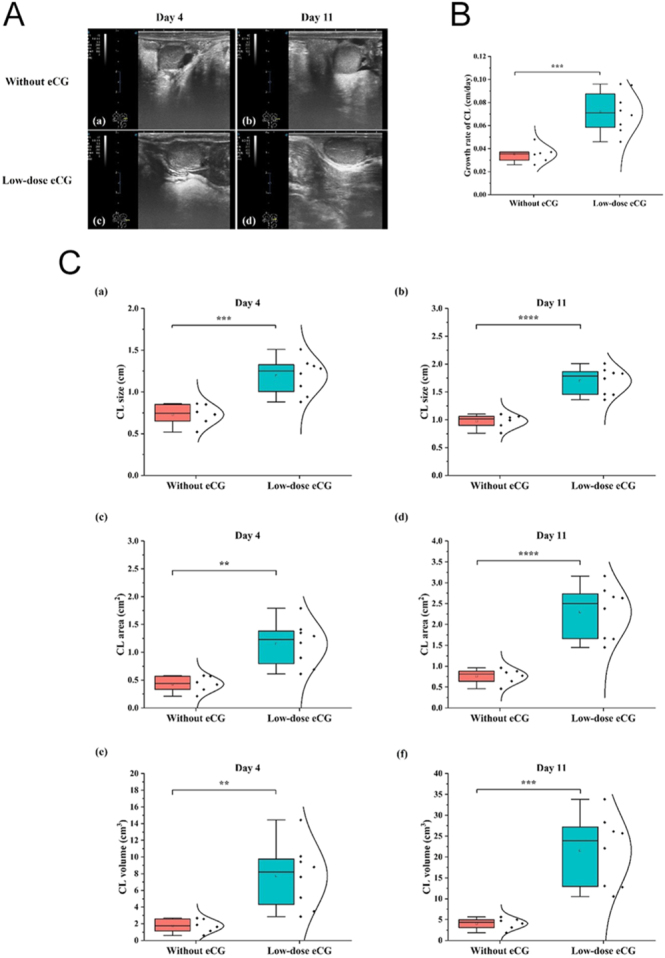
Effects of low-dose eCG treatment on corpus luteum (CL) development in cows during the luteal phase. (A) Representative ultrasonographic images of the CL on day 4 and day 11 post-ovulation in cows treated without and with low-dose eCG. (B) Comparison of CL growth rate (cm/day) between treatment groups. (C) Box plots showing CL size, area, and volume on day 4 and day 11 in cows without and with low-dose eCG treatment: (a, b) CL size (cm), (c, d) CL area (cm^2^), and (e, f) CL volume (cm^3^). Asterisks indicate statistically significant differences (**P < 0.01, ***P < 0.001, and ****P < 0.0001).

Ultrasonographic monitoring revealed distinct differences in CL development between cows treated with low-dose eCG and those without eCG administration (Fig. 4A). On both day 4 and day 11 post-ovulation, cows in the low-dose eCG group exhibited visibly larger and more developed CL structures compared to the control group (Fig. 4A(a,b,c,d)). Quantitative analysis showed that the CL growth rate was significantly higher in the low-dose eCG group than in the control group (*P* < 0.001; Fig. 4B), indicating an enhanced luteal development trajectory. CL size, area, and volume were significantly greater in cows treated with low-dose eCG at both day 4 and day 11. Specifically, CL size on day 4 and day 11 was significantly larger in the eCG group (*P* < 0.001 and *P* < 0.0001, respectively; Fig. 4C(a,b)). Likewise, CL area showed significant increases in the low-dose eCG group at both time points (*P* < 0.01 on day 4; *P* < 0.0001 on day 11; Fig. 4C(c,d)). CL volume measurements mirrored these findings. On day 4, the low-dose eCG group had significantly higher CL volumes than untreated cows (*P* < 0.01; Fig. 4C(e)), and this difference was even more pronounced by day 11 (*P* < 0.001; Fig. 4C(f)). Collectively, these results demonstrate that low-dose eCG administration post-CIDR removal significantly stimulates CL development and enhances luteal tissue growth, which may contribute to improved luteal function and reproductive outcomes.

### Hemodynamic changes/luteal blood perfusion during early and mid-luteal phases

**Figure 5:**
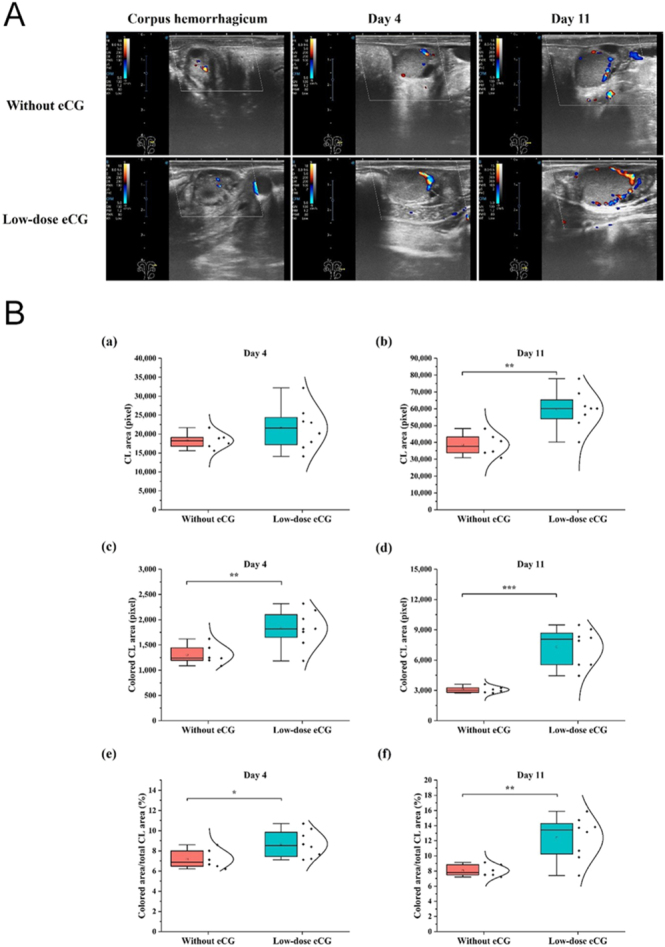
Effects of low-dose eCG treatment on corpus luteum (CL) development and hemodynamic changes in cows. (A) Representative color Doppler ultrasonographic images of the CL at the corpus hemorrhagicum stage, and on day 4 and day 11 post-ovulation, in cows without and with low-dose eCG treatment. (B) Quantitative analysis of CL area and hemodynamic changes: (a,b) CL area (pixel) on day 4 and day 11, respectively. (c,d) Colored CL area (pixel), indicating hemodynamic changes, on day 4 and 11, respectively. (e,f) Proportion of colored area to total CL area (%) on day 4 and day 11, respectively. Boxplots show median, interquartile range, individual data points, and density distribution. Asterisks indicate statistically significant differences between groups (*P < 0.05, **P < 0.01, and ***P < 0.001).

Color Doppler ultrasonography revealed greater blood perfusion in CL of cows treated with low-dose eCG compared to untreated controls on both day 4 and day 11 (Fig. 5A). Enhanced blood perfusion was evident from the increased colored areas indicating blood flow within the luteal tissue. Quantitative analysis of CL area (Fig. 5B(a,b)) demonstrated a significant enlargement in the low-dose eCG group on both day 4 (*P* < 0.01) and day 11 (*P* < 0.01). Furthermore, the colored CL area, representing blood flow, was significantly greater in the low-dose eCG group on both day 4 and day 11 (*P* < 0.01 and *P* < 0.001, respectively; Fig. 5B(c,d)). When evaluating blood perfusion relative to total CL area, the proportion of colored CL area (% of CL area) was also significantly higher in the eCG group. This difference was statistically significant on day 4 (*P* < 0.05) and even more so on day 11 (*P* < 0.01; Fig. 5B(e,f)). These findings suggest that low-dose eCG not only stimulates luteal tissue development but also markedly enhances luteal blood perfusion, which may contribute to improved luteal function and P4 production.

### Circulating E2 and P4 production

**Figure 6:**
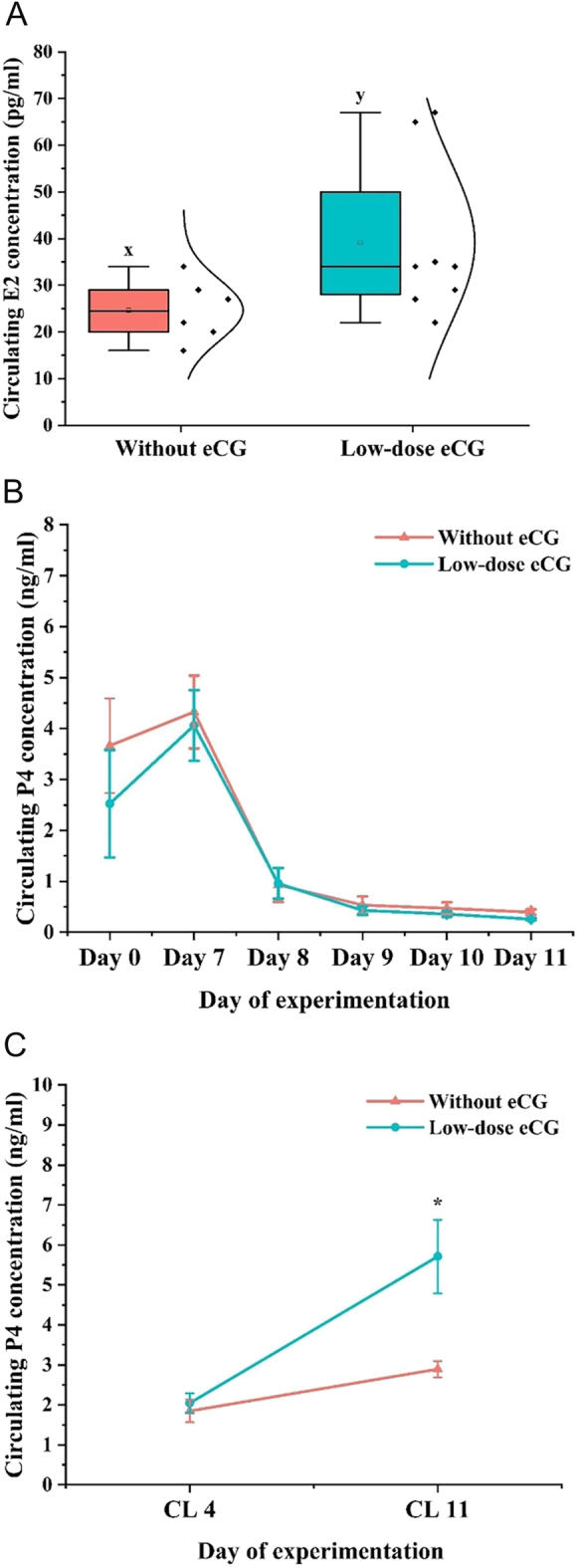
Effects of low-dose eCG treatment on circulating estradiol (E2) and progesterone (P4) concentrations in cows. (A) Box plot comparing circulating E2 concentration (pg/mL) at the preovulatory stage between cows without and with low-dose eCG treatment. (B) Changes in circulating P4 concentrations (ng/mL) in both groups. (C) Circulating P4 concentrations (ng/mL) on day 4 (CL4) and 11 (CL11) post-ovulation in cows treated without and with low-dose eCG. Different superscript (x,y) letters indicate a tendency toward significance (0.05 < *P* ≤ 0.01), and asterisks denote statistically significant differences (*P < 0.05).

The administration of low-dose eCG significantly influenced circulating steroid hormone concentrations during the preovulatory stage and the luteal phase (Fig. 6). Plasma E2 concentration on the day of standing estrus tended to be higher in the low-dose eCG group compared to the control group without eCG (*P* = 0.07; Fig. 6A). The median E2 concentration in the low-dose eCG group was notably elevated, indicating an enhanced follicular response to hormonal stimulation. Longitudinal assessment of circulating P4 levels showed no significant difference between groups from day 0 to day 11; however, both groups peaked on day 7 before declining (Fig. 6B). Notably, when analyzing P4 concentration based on CL age, cows treated with low-dose eCG exhibited significantly higher P4 levels on day 11 of the luteal phase (CL11) compared to the group without eCG (*P* < 0.05; Fig. 6C). This suggests a stimulatory effect of eCG on luteal function in the mid-luteal phase. These results demonstrate that low-dose eCG promotes increased pre-ovulatory E2 secretion and augments luteal P4 production later in the cycle, supporting improved ovarian function.

### Luteal angiogenesis and vascular remodeling gene expression

**Figure 7:**
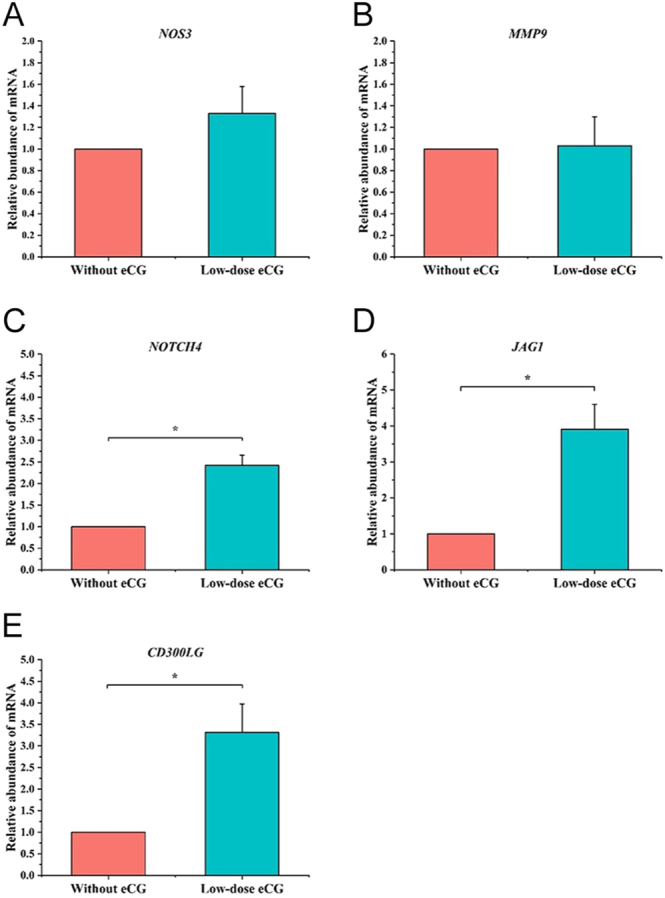
Effects of low-dose eCG treatment on mRNA expression of *NOS3*, *MMP9*, *NOTCH4*, *JAG1*, and *CD300LG* in luteal tissue samples from cows. Relative mRNA expression levels of (A) *NOS3*, (B) *MMP9*, (C) *NOTCH4*, (D) *JAG1*, and (E) *CD300LG* in luteal tissues collected from cows treated without and with low-dose eCG. Data are presented as mean ± SD. Asterisks (*) indicate statistically significant differences (*P* < 0.05).

To evaluate the effects of low-dose eCG on molecular markers of luteal angiogenesis and vascular remodeling, the relative mRNA expression of key genes in the CL was analyzed (Fig. 7). There were no significant differences in the expression of *NOS3* (Fig. 7A) or *MMP9* (Fig. 7B) between the control and low-dose eCG-treated groups, although a numerical increase in *NOS3* expression was observed following eCG administration. The expression of *NOTCH4* was significantly higher in the low-dose eCG group compared to the group without eCG treatment (*P* < 0.05; Fig. 7C). Similarly, a marked upregulation of *JAG1*, a key ligand in the Notch signaling pathway, was detected in the low-dose eCG group (*P* < 0.05; Fig. 7D). These findings suggest that eCG promotes the activation of Notch-mediated signaling during luteal development. The transcript level of *CD300LG*, a gene associated with endothelial integrity and vascular permeability, was significantly elevated in the low-dose eCG group (*P* < 0.05; Fig. 7E), indicating enhanced vascular adaptation in response to eCG stimulation. Overall, these results indicate that low-dose eCG enhances the expression of genes involved in vascular development and remodeling within the CL, particularly those associated with Notch signaling and endothelial function.

### Correlation analysis among evaluated traits

**Figure 8:**
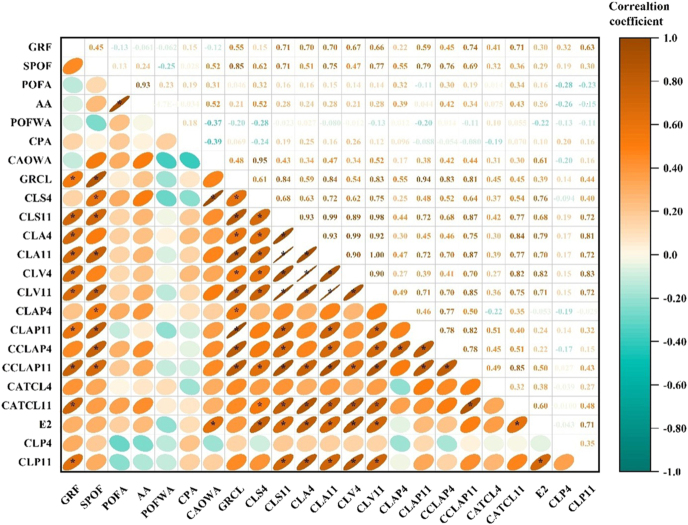
Visualized correlation matrix of follicular, luteal, and hormonal parameters in cows. The plot depicts Pearson correlation coefficients among key follicular development metrics, luteal morphological and hemodynamic parameters, and circulating hormone concentrations in cows. The strength and direction of correlations are represented by both color and shape: orange ovals indicate positive correlations, blue-green ovals indicate negative correlations, and the shape elongation reflects the magnitude of the correlation. Asterisks (*) denote statistically significant correlations (*P < 0.05). The color scale ranges from −1 to 1, with values near ±1 indicating stronger linear relationships. GRF, growth rate of follicle (mm/day); SPOF, size of POF (mm); POFA, POF area (pixel); AA, antral area (pixel); POFWA, POF area without antrum (pixel); CPA, colored POF area (pixel); CAOWA, colored area/POF area without antrum (%); GRCL, growth rate of CL (cm/day); CLS4, CL size on day 4 (cm); CLS11, CL size on day 11 (cm); CLA4, CL area on day 4 (cm^2^); CLA11, CL area on day 11 (cm^2^); CLV4, CL volume on day 4 (cm^3^); CLV11, CL volume on day 11 (cm^3^); CLAP4, CL area (pixel) on day 4; CLAP11, CL area (pixel) on day 11; CCLAP4, colored CL area (pixel) on day 4; CCLAP11, colored CL area (pixel) on day 11; CATCL4, colored area/total CL area (pixel) on day 4; CATCL11, colored area/total CL area (pixel) on day 11; E2, estradiol levels (pg/mL); CLP4, P4 levels on day 4 (ng/mL); CLP11, P4 levels on day 11 (ng/mL).

A correlation analysis was conducted to evaluate the relationships among the studied traits (Fig. 8). Strong positive correlations (*r* > 0.80) were observed among SPOF and POFA (*r* = 0.93), CLS11 and CLA4 (*r* = 0.93), and CLS11 and CLA11 (*r* = 0.90), indicating that follicle size is closely linked with subsequent luteal development. GRCL also correlated strongly with CLS11 (*r* = 0.83), CLV4 (*r* = 0.82), and CLA4 (*r* = 0.85), underscoring its importance as an indicator of luteal function. Moderate positive correlations (0.50 < *r* < 0.80) were found between GRF and SPOF (*r* = 0.66), POFA (*r* = 0.71), and CLS11 (*r* = 0.71), reflecting coordinated regulation between follicular growth and luteal outcomes. In contrast, CPA showed weak negative associations with GRF (*r* = −0.39) and POFA (*r* = −0.28). Together, these correlations highlight the integrated relationships among follicular growth, ovulatory follicle size, luteal development, and hormone production, emphasizing the role of eCG in enhancing ovarian function.

## Discussion

This study demonstrates that low-dose eCG supplementation during short-term estrous synchronization enhances DF growth, CL development, luteal hemodynamics, steroidogenesis, and angiogenic gene expression in White Lamphun cows. eCG, possessing both FSH- and LH-like activities, promotes follicular recruitment, granulosa cell proliferation, and E2 synthesis (Baruselli *et al.* 2004, Ribas *et al.* 2018, Ferraz *et al.* 2019, Bottino *et al.* 2021). Consequently, cows receiving eCG showed higher follicular growth rates and larger POFs than controls, consistent with reports in *Bos indicus* breeds (Bó & Baruselli 2014, Bó *et al.* 2018). Larger, well-vascularized follicles provide a superior intrafollicular environment, enhancing oocyte competence and luteinization, which in turn increase CL volume and P4 secretion (Acosta & Miyamoto 2004, Herzog *et al.* 2010, Abdelnaby *et al.* 2018, Couto *et al.* 2023). These outcomes align with earlier findings that eCG improves ovulation rate, CL size, and P4 concentration (Barreiros *et al.* 2014, Teixeira *et al.* 2020). Overall, the improvements observed in White Lamphun cows parallel those reported in other *Bos indicus* populations, confirming the physiological and practical value of eCG in synchronization and fixed-time AI programs under tropical conditions (Bó *et al.* 2003, Baruselli *et al.* 2004).

Low-dose eCG supplementation significantly enhanced CL development, as shown by greater CL diameter, area, and volume on days 4 and 11 post-ovulation, and a faster CL growth rate compared with controls. These results indicate that eCG promotes a more rapid and robust luteal response during the early luteal phase. The LH-like activity of eCG administered at CIDR removal likely facilitates follicular cell differentiation into luteal cells, producing a larger and more hemodynamically active CL (Núñez-Olivera *et al.* 2014, Fátima *et al.* 2018, Abdelnaby *et al.* 2021). Given the reported half-life of eCG in cattle (46–62 h), its effects observed at day 11 are likely indirect, mediated through improved preovulatory follicular growth and blood perfusion (Acosta & Miyamoto 2004, Herzog *et al.* 2010, Lonergan *et al.* 2016). Larger CLs corresponded with increased P4 concentrations, consistent with previous findings linking CL size with luteal steroidogenic capacity (Gómez-Seco *et al.* 2017, Monteiro *et al.* 2021, El Azzi *et al.* 2025). Similarly, eCG-treated cows exhibited higher P4 levels by day 11, aligning with earlier reports of enhanced luteal function following eCG treatment (Teixeira *et al.* 2020, Véliz-Deras *et al.* 2020). These findings are particularly relevant for *Bos indicus* breeds such as White Lamphun cattle, which typically exhibit smaller follicles and weaker luteal responses to endogenous LH (Sartori *et al.* 2010). Thus, eCG offers a practical approach to strengthen luteal function and fertility in tropical cattle systems (Neglia *et al.* 2015, Fournier *et al.* 2021).

Color Doppler ultrasonography revealed significantly greater colored and colored/total areas in both POF and CL of cows treated with low-dose eCG, indicating enhanced angiogenesis and blood perfusion essential for ovarian function (Bollwein *et al.* 2002). Increased luteal blood flow on days 4 and 11 post-ovulation reflects improved blood perfusion, consistent with findings that luteal perfusion is closely associated with P4 output and fertility (Herzog *et al.* 2010, Lüttgenau & Bollwein 2014). Enhanced follicular vascularity in the eCG group suggests greater endocrine competence, supporting efficient estradiol synthesis and the preovulatory LH surge (Acosta *et al.* 2003, Sá Filho *et al.* 2010, Barreiros *et al.* 2014, Neto *et al.* 2024). These hemodynamic improvements corresponded with larger CLs and elevated plasma P4 levels, reinforcing that blood flow is a more accurate indicator of luteal activity than CL size alone (Herzog *et al.* 2010). The increased blood perfusion observed is likely mediated by eCG-induced angiogenic signaling, including the stimulation of VEGF and activation of Notch (*NOTCH4*, *JAG1*) and CD300LG pathways (Sousa *et al.* 2016, Fátima *et al.* 2018, Kfir *et al.* 2018). Collectively, these structural and molecular changes demonstrate that eCG enhances ovarian vascular function, improving follicular competence, luteal activity, and overall fertility.

Preovulatory E2 concentrations were significantly higher in the low-dose eCG group, reflecting enhanced follicular activity. This aligns with the observed increase in DF size and the role of eCG’s FSH- and LH-like activity in stimulating granulosa and theca cell steroidogenesis, thereby promoting E2 synthesis (Tortorella *et al.* 2013, Ribas *et al.* 2018, Núñez-Olivera *et al.* 2020). Elevated E2 is essential for inducing the LH surge and optimizing ovulation and uterine receptivity (Esparza-Harris 2017). Although early luteal P4 levels (day 4) did not differ significantly, cows treated with eCG showed markedly higher P4 by day 11, consistent with larger and more vascularized CLs (Lonergan 2011, Salehnia & Zavareh 2013). Adequate luteinization of granulosa and theca cells, supported by eCG’s LH-like action, enhances luteal steroidogenic capacity (Abedel-Majed *et al.* 2019). Sustained mid-luteal P4 is crucial for embryo development, maternal recognition of pregnancy, and immune modulation (Lonergan *et al.* 2016, Martins *et al.* 2018). Collectively, these results confirm that eCG supplementation optimizes both follicular E2 production and luteal P4 secretion, ensuring a balanced endocrine environment that supports conception and early pregnancy, particularly in *Bos indicus* breeds such as White Lamphun cattle.

Low-dose eCG supplementation significantly upregulated angiogenesis-related genes, including *NOTCH4*, *JAG1*, and *CD300LG*, in the CL on day 11 post-ovulation, while *NOS3* and *MMP9* showed no significant change, suggesting selective activation of angiogenic pathways. The Notch signaling pathway, mediated through *NOTCH4* and its ligand *JAG1*, regulates endothelial communication and vascular remodeling (Uyttendaele *et al.* 2001, Kfir *et al.* 2018, Souilhol *et al.* 2022). Their upregulation in the eCG group aligns with enhanced luteal blood perfusion detected via color Doppler imaging, reflecting improved capillary sprouting and vessel stabilization (Gridley 2001, Iso *et al.* 2003, Sainson *et al.* 2005). *CD300LG*, an endothelial adhesion molecule associated with vascular permeability and tissue-specific angiogenesis (Takatsu *et al.* 2006, Liu *et al.* 2020), was also upregulated, indicating potential roles in immune modulation and vascular integrity within the developing CL. Although *NOS3* and *MMP9*, involved in nitric oxide-mediated vasodilation and extracellular matrix remodeling (Tamanini *et al.* 2003, Rundhaug 2005, Nath & Maitra 2019), were not significantly altered, their expression may vary temporally or act in earlier luteal stages. Together, these results suggest that eCG enhances luteal angiogenesis primarily through activation of the NOTCH–JAGGED and CD300LG pathways, consistent with the observed increases in CL size, perfusion, and P4 synthesis, thereby supporting improved luteal function and fertility in White Lamphun cattle.

Correlation analysis revealed strong positive associations among key ovarian parameters, including follicular growth rate, POF size, CL area and volume, blood perfusion indices, and circulating E2 and P4 levels. These interrelationships highlight the coordinated regulation of follicular development, ovulation, luteal function, and endocrine activity under eCG-synchronized protocols. A higher follicular growth rate correlated with larger POF area, consistent with findings that rapid follicular growth promotes DF competence and E2 synthesis via gonadotropin-stimulated aromatase activity (Varughese *et al.* 2014, Dewailly *et al.* 2016, François *et al.* 2017). Larger ovulatory follicles were associated with greater CL size and volume, supporting reports that follicular health influences subsequent luteal capacity and P4 production (Pinaffi *et al.* 2015, López-Gatius *et al.* 2022). Similarly, CL blood perfusion correlated strongly with plasma P4 levels, reinforcing that blood flow is a major determinant of steroidogenic activity (Bollwein *et al.* 2012, Kaya *et al.* 2017). Color Doppler parameters of POFs and CLs were closely linked to their respective hormone outputs, indicating that vascular support underlies both E2 and P4 synthesis (Ishak *et al.* 2017, Pandey *et al.* 2018). Collectively, these data confirm that ovarian structure, blood perfusion, and endocrine function form an integrated system responsive to eCG stimulation. Such correlations provide practical value for predicting reproductive competence using non-invasive Doppler-based indicators.

Although this study provides strong physiological and hemodynamic evidence of the carry-over effects of eCG on both the follicular and luteal phases, further investigation is needed to clarify the underlying molecular mechanisms. Future research will explore upstream regulatory pathways, including VEGF, pentraxin 3 (PTX3), and endothelin-1 (EDN1), as well as temporal gene expression dynamics during luteal development, to elucidate how eCG modulates angiogenic and steroidogenic processes in *Bos indicus* cattle.

## Conclusion

The present study demonstrates that low-dose eCG supplementation during a short-term estrous synchronization protocol significantly improves reproductive outcomes in indigenous White Lamphun cows. eCG administration enhanced DF growth and promoted more robust CL development. These improvements were accompanied by enhanced luteal blood perfusion, as assessed by color Doppler ultrasonography, and elevated P4 levels in the mid-luteal phase. Furthermore, the upregulation of angiogenesis- and vascular remodeling-related genes (*NOTCH4*, *JAG1*, and *CD300LG*) in luteal tissue highlights the molecular basis for improved luteal function following eCG treatment. Collectively, these findings indicate that low-dose eCG not only optimizes follicular and luteal physiology but also contributes to the endocrine environment favorable for successful reproduction. This protocol may serve as an effective reproductive management strategy to improve fertility in indigenous White Lamphun cattle, particularly under tropical and resource-limited production systems.

## Declaration of interest

The authors declare that there is no conflict of interest that could be perceived as prejudicing the impartiality of the work reported.

## Funding

This research project is supported by the Faculty of Agriculture, Chiang Mai University.

## Author contribution statement

JJ and TM were responsible for conceptualization, data curation, visualization, and writing the original draft. JJ was responsible for methodology, project administration, supervision, and funding acquisition. PY, MS, AS, WB, ND, JJ, and TM were responsible for investigation. PY, MS, AS, WB, ND, and JJ provided resources. AT, SB, CJL, YZ, JJ, and TM were responsible for writing review and editing. All authors have read and agreed to the published version of the manuscript.

## Data availability

Data are contained within the article and will be available upon request.

## Institutional review board statement

All procedures involving animal subjects were carried out under the Institutional Animal Care and Use Committee (IACUC) and approved by the Institutional Animal Care and Use Committee of the Faculty of Agriculture, Chiang Mai University, Chiang Mai, Thailand (Approval No. RAGIACUC028/2567, 30 October 2024).
